# *In silico* prediction of the metabolism of *Blastocrithidia nonstop*, a trypanosomatid with non-canonical genetic code

**DOI:** 10.1186/s12864-024-10094-8

**Published:** 2024-02-16

**Authors:** Fred R. Opperdoes, Kristína Záhonová, Ingrid Škodová-Sveráková, Barbora Bučková, Ľubomíra Chmelová, Julius Lukeš, Vyacheslav Yurchenko

**Affiliations:** 1 de Duve Institute, Université catholique de Louvain, Brussels, Belgium; 2 Life Science Research Centre, Faculty of Science, University of Ostrava, Ostrava, Czechia; 3 Institute of Parasitology, Biology Centre, Czech Academy of Sciences, České Budějovice, Czechia; 4 Department of Parasitology, Faculty of Science, Charles University, BIOCEV, Vestec, Czechia; 5 Division of Infectious Diseases, Department of Medicine, University of Alberta, Edmonton, Canada; 6 Department of Biochemistry, Faculty of Natural Sciences, Comenius University, Bratislava, Slovakia; 7 Faculty of Science, University of South Bohemia, České Budějovice, Czechia

**Keywords:** *Blastocrithidia*, *In silico*, Metabolic predictions, Trypanosomatid, Non-canonical genetic code

## Abstract

**Background:**

Almost all extant organisms use the same, so-called canonical, genetic code with departures from it being very rare. Even more exceptional are the instances when a eukaryote with non-canonical code can be easily cultivated and has its whole genome and transcriptome sequenced. This is the case of *Blastocrithidia nonstop*, a trypanosomatid flagellate that reassigned all three stop codons to encode amino acids.

**Results:**

We *in silico* predicted the metabolism of *B. nonstop* and compared it with that of the well-studied human parasites *Trypanosoma brucei* and *Leishmania major*. The mapped mitochondrial, glycosomal and cytosolic metabolism contains all typical features of these diverse and important parasites. We also provided experimental validation for some of the predicted observations, concerning, specifically presence of glycosomes, cellular respiration, and assembly of the respiratory complexes.

**Conclusions:**

In an unusual comparison of metabolism between a parasitic protist with a massively altered genetic code and its close relatives that rely on a canonical code we showed that the dramatic differences on the level of nucleic acids do not seem to be reflected in the metabolisms. Moreover, although the genome of *B. nonstop* is extremely AT-rich, we could not find any alterations of its pyrimidine synthesis pathway when compared to other trypanosomatids. Hence, we conclude that the dramatic alteration of the genetic code of *B. nonstop* has no significant repercussions on the metabolism of this flagellate.

**Supplementary Information:**

The online version contains supplementary material available at 10.1186/s12864-024-10094-8.

## Introduction

Trypanosomatids (family Trypanosomatidae) are a species-rich, evolutionary and ecologically diverse group of flagellated protists belonging to the class Kinetoplastea (phylum Euglenozoa) [1]. As virtually omnipresent parasites of invertebrate and vertebrate hosts, their diversity appears to be vast [2]. The best-known members of this group are *Trypanosoma brucei* and *Leishmania* spp., causative agents of the African sleeping sickness and leishmaniasis, respectively [3–5]. Due to their genetic tractability and intense studies, they serve as the model eukaryotic species [6–8]. Their metabolism has been mapped to a considerable detail [9–11] and functional annotations of about 9,000 protein-coding genes are also well-advanced [12–16].

However, trypanosomatids are comprised of 24 genera, many of which have been established based on phylogenetic analyses only relatively recently [1, 17]. Hence, except the well-studied disease-causing members of the genera *Trypanosoma*, *Leishmania*, and *Phytomonas*, very little is known about the cellular and molecular features of the remaining trypanosomatid lineages. The ongoing efforts aim to assemble and annotate the genomes of species representing the known diversity of this prominent group of parasitic flagellates [18, 19]. Despite their relative morphological uniformity, members of distinct genera contain a rather diverse collection of metabolic pathways from the expanded, contracted, and/or specialized protein families [20–23]. Metabolic flexibility underlies the diverse and frequently complex parasitic lifestyles, as these protists are capable of infecting virtually every eukaryote, ranging from other protists to mammals [2, 24].

From the genomic perspective, one group of trypanosomatids stands particularly out. It is the morphologically and ultra-structurally inconspicuous but genetically exceptional genus *Blastocrithidia* [25], which has recoded all three stop codons in its nuclear genome into sense codons, thus representing one of the most prominent departures from the canonical genetic code [26]. An iconic species of this genus, *Blastocrithidia nonstop* was isolated from the true bug (shieldbug) *Eysarcoris aeneus* in Czechia a few years ago. Similarly to many other cosmopolitan trypanosomatids (the species under study was also documented in Asia, Africa, South America, and the Australasian realm), *B. nonstop* has a low host specificity as it can infect at least 14 different species of true bugs belonging to eight families. Notably, representatives of the closest phylogenetic kin of *Blastocrithidia*, *Obscuromonas* spp. have standard genetic code and, based on the phylogenetic reconstructions, the divergence of these two genera happened relatively recently [25]. Moreover, novel features associated with a massively altered genetic code of *B. nonstop*, namely the truncation of the anticodon stem of the transfer RNA dedicated to the readthrough of the in-frame UGA codon and the overall non-random distribution of the in-frame stop codons across the protein-coding genes [27] qualify this flagellate as an interesting model organism for studies of the departures from the standard genetic code [28]. Although it is unclear what triggered the wholesale recoding of the stop into sense codons in *B. nonstop*, it was proposed that the unusual AT-richness of its nuclear genome may be one of the key factors. It was shown before that in a subset of genes encoding Krebs cycle enzymes, UAG and UAA coding for Glu were significantly depleted [26]. On the whole-genome scale, the frequency of in-frame stop codons in protein-coding genes negatively correlates with the abundance of the corresponding proteins in this species [27]. We hypothesized that because of this, some metabolic pathways may run slowly or some pathways (or their parts) may get ablated reflecting a burden imposed on their components by the accumulated in-frame stop codons.

Thanks to the availability of a high-quality nuclear genome assembly of *B. nonstop* [27], we could predict its overall metabolism *in silico*. As an important disclaimer, please note that many of the predictions presented below are just computational and, as such they will need to be validated experimentally in the future. At the same time, we believe that our analyses provide a strong foundation for further research into biology of this truly fascinating species.

## Results and discussion

### Glycosomes and acidocalcisomes

**Fig. 1:**
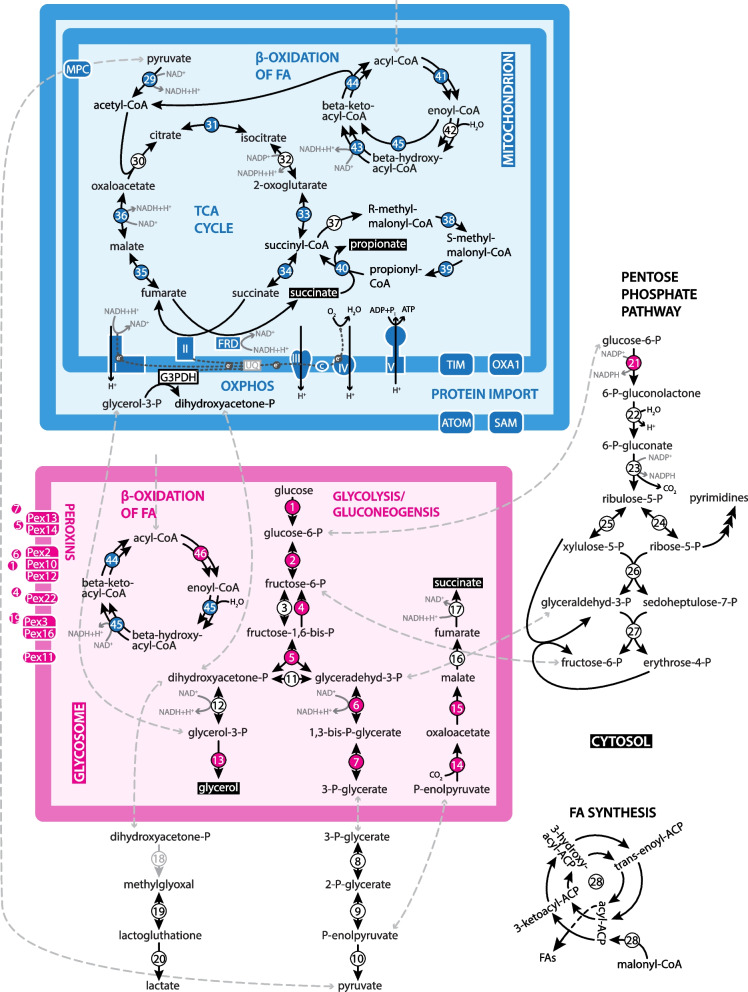
Metabolic pathways present in *B. nonstop*. The end-products are shown in white font on black background. Numbers in colors represent proteins with predicted targeting signal (mitochondrial, blue; PTS1/2, magenta; no signal; white). Numbers and arrows in light-grey represent enzymes that were not identified. Numbers and abbreviations are explained in Table 1

Proteins involved in biogenesis of peroxisomes (peroxins) [29, 30] were all detected in the *B. nonstop* proteome (Fig. 1; Table 1). In both, trypanosomatids and diplonemids, the peroxisome catalyzes a part of the glycolytic pathway and, hence, is named the glycosome [31, 32]. In *B. nonstop*, the situation is not different. Typical glycolytic enzymes contain a peroxisomal targeting signal (PTS) of type 1 or 2 [29, 33] (Table 1). In addition to the glycolytic enzymes, purine salvage pathway, pyrimidine biosynthesis, and oxidative stress protection are predicted to be present in the *B. nonstop* glycosomes (see below). As in other euglenozoans [34], the pentose-phosphate pathway can have a dual localization in the cytosol and glycosomes, since at least the first enzyme of the pathway has a clear PTS1 signal (Table 1). Thus, *B. nonstop* is predicted to harbor *bona fide* peroxisomes with metabolic capacity similar to glycosomes of other well-studied trypanosomatids (Table S1) [34]. The presence of glycosomes in *B. nonstop* was validated experimentally by immunofluorescence microscopy with anti-TIM (visualizing glycolytic triosephosphate isomerase [35] (Table 1)) and anti-MVAK (visualizing phosphomevalonate kinase, an enzyme of the isoprenoid biosynthesis pathway [36]) (Fig. S1). Specificity of both antibodies has been previously validated in phylogenetically-distant *Leishmania* and *Trypanosoma* spp. [36–38] suggesting that they are suitable for studies in diverse trypanosomatids. The patterns of organelles recognized by these two antibodies are different likely reflecting the heterogeneity of glycosomes in trypanosomatids [39].

**Table 1:**
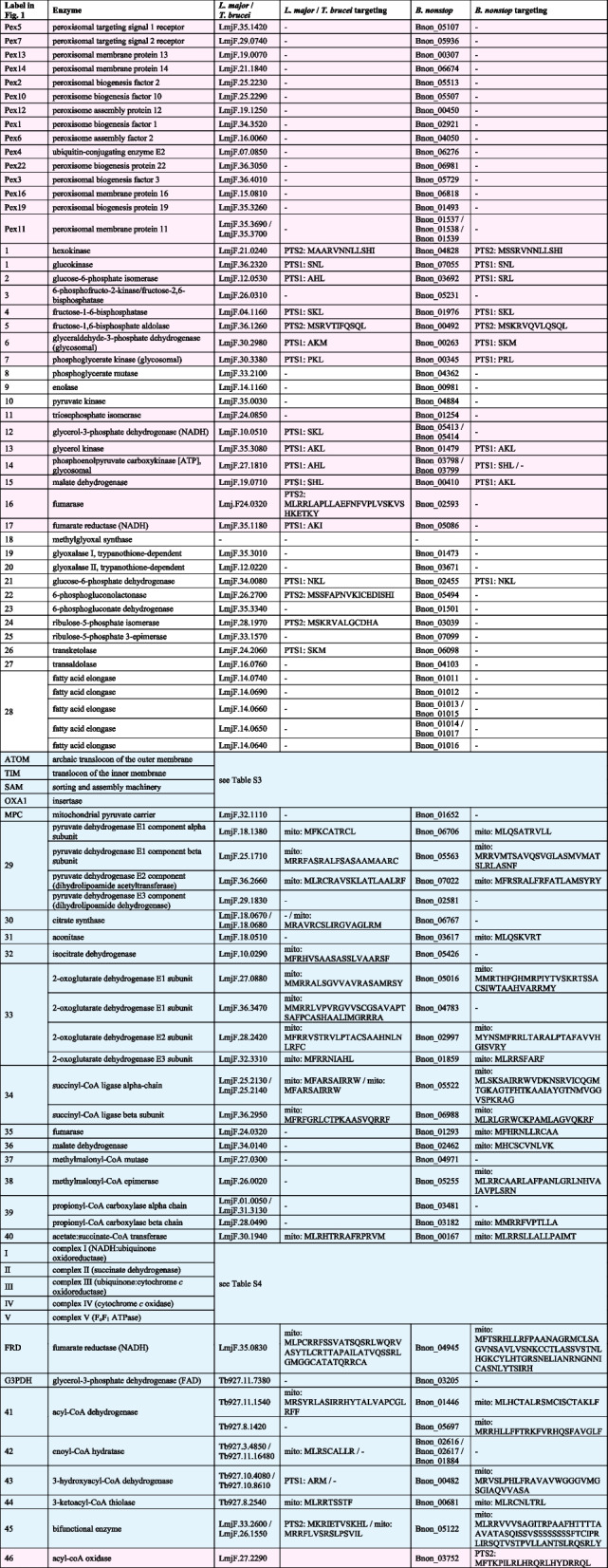
List of selected enzymes involved in metabolism of *B. nonstop*. Pink and blue backgrounds indicate putative glycosomal and mitochondrial localization, respectively

None of the dixenous (with two hosts in the life cycle [40]) trypanosomatids (*Leishmania, Trypanosoma*, and *Phytomonas* spp.) possess a gene for the typical peroxisomal marker enzyme catalase, while it has been acquired by horizontal gene transfer (HGT) and retained by some monoxenous (with one host in the life cycle) Leishmaniinae and a few other trypanosomatid lineages [41]. *Blastocrithidia nonstop* belongs to the latter group and its catalase is different from the homologs in other trypanosomatid species [42, 43].

Acidocalcisomes of *B. nonstop* are predicted to be similar to their kin in other trypanosomatids [44–46] (Table S2). These organelles function as storage of cations and phosphorus, and are involved in calcium homeostasis, maintenance of intracellular pH homeostasis, and osmoregulation [44]. Besides trypanosomes, where they were first characterized [47, 48], these organelles were also identified in other protists, for example, in the apicomplexan parasites *Toxoplasma gondii* and *Plasmodium falciparum*, or the green alga *Chlamydomonas reinhardtii* [49–51].

### Mitochondrion

As in other kinetoplastids, the mitochondrion of *B. nonstop* is predicted to harbor hallmark proteins of the translocation machinery, i.e., ATOM (archaic translocase of the outer membrane) and TIM (translocase of the inner membrane) [52]. Components of the SAM (sorting and assembly machinery) and Oxa1 insertase necessary for inserting transmembrane proteins to the outer and inner membrane, respectively, were also identified (Fig. 1; Table 1; Table S3). Genes for the standard mitochondrial enzymes and metabolic pathways were all identified (see below; Tables S3-S5).

#### Krebs cycle

A complete set of the predicted Krebs (tricarboxylic acid/TCA) cycle enzymes (Fig. 1; Table 1) potentially enables *B. nonstop* to run a full cycle or use separate reactions for other purposes than for complete oxidation of mitochondrial substrates [53, 54]. A replacement of the eukaryotic-type succinyl-coenzyme A (CoA) synthetase for the bacterial-type succinyl-CoA ligase, which is a tetramer composed of two α and β subunits found in kinetoplastids, may allow the reaction to function in both catabolic and anabolic directions. Most predicted TCA enzymes are endowed with a mitochondrial targeting signal (MTS) at their N-termini. There are five predicted malate dehydrogenases – one mitochondrial, one glycosomal, and three cytosolic isoenzymes (Table 1; Table S5). As in most trypanosomatids, the single mitochondrial isocitrate dehydrogenase is predicted to be an NADP^+^-dependent enzyme suggesting that this enzyme in *B. nonstop* is also involved in the TCA cycle and enables reductive carboxylation rather than the complete oxidation of pyruvate to CO_2_ and H_2_O [55, 56].

#### Respiratory chain and oxidation of mitochondrial NADH

The mitochondrial genome (termed kinetoplast DNA) of *B. nonstop* is predicted to encode the same set of genes that is present in most other trypanosomatids [57, 58]. Moreover, the nucleus-encoded subunits of complex I (NADH:ubiquinone oxidoreductase), complex II (succinate dehydrogenase), complex III (ubiquinone:cytochrome *c* oxidoreductase), complex IV (cytochrome *c* oxidase), complex V (F_o_F_1_ ATPase) are all appear to be present, as well as cytochromes *b*, *c*, and *c1* [23, 59–63] (Table S4). The alternative NADH dehydrogenase (NDH2) [64] and the alternative oxidase [65, 66] are conspicuously absent.

Despite the predicted absence of enzymes involved in the biosynthesis of quinoid ring structure (UbiE, UbiF, UbiG and UbiH) of ubiquinone (UQ) and its prenyl-side chain (solanesyl-diphosphate synthase) [67, 68] that are all present in most trypanosomatids (Table S4), the substrate-stimulated oxygen consumption appears intact and sensitive to malonic acid (which inhibits complex II) in *B. nonstop* (Fig. S2). This suggests that this parasite is unable to form its own UQ resembling the situation encountered in the free-living kinetoplastid *Bodo saltans*, which lost the same set of enzymes [69]. In the latter case, it was hypothesized that the phagotrophic lifestyle of a bodonid that relies on bacteria providing a continuous supply of UQ [70] may have facilitated the loss of the UQ biosynthetic pathway enzymes. It remains to be investigated further what caused this wholesale loss in *B. nonstop.* Of note, the mentioned above presence of several obviously functional electron-transfer proteins carrying electrons from a reduced substrate to the electron acceptor UQ is another piece of evidence that supports the functionality of electron transport through an external UQ source. The possibility that *B. nonstop* would utilize rhodoquinone (RQ) rather than UQ as a carrier of the reducing equivalents between the different respiratory chain complexes is highly unlikely. Genes encoding proteins responsible for the formation of RQ in a handful of anaerobic bacteria and eukaryotes (such as RQ biosynthesis methyltransferase or certain enzymes of the kynurenine pathway [71]) were not detected in *B. nonstop* or any other trypanosomatid.

A possible alternative pathway for the re-oxidation of mitochondrial NADH in *B. nonstop* relies on the presence of a mitochondrial NADH-dependent fumarate reductase (FRD) (Fig. 1). Two isoenzymes, of which one carries a clear MTS, were predicted in the genome of this flagellate (Table 1). These NADH-FRD enzymes appear different from the mitochondrial RQ-dependent fumarate reductases of anaerobic eukaryotes [72, 73]. The involvement of glycosomal and mitochondrial NADH-FRDs in the production of succinate as the metabolic end-product has been described in other trypanosomatids [74, 75] and may be functional in *B. nonstop* as well (Fig. 1).

The predicted presence of the methylmalonyl-CoA pathway enzymes (methylmalonyl-CoA epimerase, methylmalonyl-CoA mutase, and propionyl-CoA carboxylase, two of which carry predicted MTSs) along with acetate:succinate-CoA transferase that may also act on propionic acid [76] (Fig. 1; Table 1; Table S5), suggests that intracellular succinate is converted to propionate *via* methylmalonyl-CoA pathway in *B. nonstop*. The excretion of propionic acid as an end-product of metabolism may be advantageous for a parasite because it results in the production of one ATP molecule *per* one molecule of propionate produced by substrate phosphorylation [77]. Whether or not propionic acid is the end-product of the *B. nonstop* metabolism under anaerobic or microaerobic conditions remains to be verified experimentally. In this respect, it is important to note that at least one trypanosomatid, *Vickermania ingenoplastis*, has been reported to excrete propionic acid in the absence of oxygen [78].

### Amino acid metabolism

#### Alanine

Both D-alanine, an amino acid found in the outer membranes of gram-negative bacteria [79], and L-alanine can possibly be utilized by *B. nonstop*. As in most other trypanosomatids [69], alanine racemase may convert D-alanine into its L-enantiomer, which can be further converted to pyruvate by the cytosolic alanine aminotransferase. Genes for all these enzymes were predicted in *B. nonstop* genome (Table S6).

#### Aspartic acid and asparagine

Aspartate is *trans*-aminated to oxaloacetate by a mitochondrial aspartate aminotransferase of narrow substrate specificity and subsequently fed into the TCA cycle [80]. Aspartate may also be formed by deamidation from asparagine by asparaginase and, subsequently, converted to fumarate *via* the purine-nucleotide cycle [81]. The genes encoding enzymes of this cycle, i.e., adenylosuccinate synthase, adenylosuccinate lyase, and AMP-deaminase are present in all kinetoplastids including *B. nonstop*. While African trypanosomes and *Phytomonas* spp. lack asparaginase, it was readily identified in the analyzed flagellate (Table S6).

#### Arginine and cysteine

Arginine is an important intermediate of the urea cycle that can serve as a substrate for the formation of polyamines [82]. Out of all kinetoplastids, only monoxenous Leishmaniinae [83] have the capacity to synthesize arginine from citrulline and aspartate [69] and *B. nonstop* appears no exception from this rule. A more detailed account of the metabolism of this amino acid is given below in the section “Urea cycle, polyamine biosynthesis, and energy storage”.

Cysteine is converted *via* mercaptopyruvate to pyruvate, which is further oxidized to acetyl-CoA by the pyruvate dehydrogenase complex (Fig. 1; Table 1). While a specific cysteine transaminase was not predicted in the genome of analyzed species, *B. nonstop* and all other kinetoplastids are equipped with a mercaptopyruvate sulfurtransferase that can convert cyanide and mercaptopyruvate to pyruvate and thiocyanate [84] (Table S6). Cysteine is either synthesized *de novo* from serine [85] or formed from homocysteine in the *trans*-sulfuration pathway *via* cystathionine-beta-synthase and cystathionine-γ-lyase [86]. Similarly to Leishmaniinae, *B. nonstop* is predicted to employ the latter pathway (Table S6).

#### Glutamate and glutamine

Since glutamate belongs to the most abundant amino acids in the insect midgut, trypanosomatids use it as a major source of carbon and ammonia [87]. It is oxidatively deaminated to the TCA cycle intermediate 2-oxoglutarate by NAD-linked glutamate dehydrogenase, an enzyme that is present in all kinetoplastids, including *B. nonstop* (Table S6). *Trypanosoma cruzi*, Leishmaniinae, and now *B. nonstop* all possess an additional glutamate dehydrogenase gene that encodes an isofunctional cytosolic NADP-dependent enzyme [69]. The corresponding gene was probably acquired *via* HGT from a gamma-proteobacterium prior the radiation of trypanosomatids, and was subsequently lost in *Phytomonas* spp. and the African trypanosomes. The function of this enzyme in trypanosomatids remains to be elucidated [88].

Glutamate and glutamine are formed by glutamine oxoglutarate aminotransferase and glutamine synthetase, respectively. As in all other trypanosomatids, only glutamine synthetase is predicted to be present in *B. nonstop* (Table S6). Because glutaminase is absent, this parasite likely cannot utilize glutamine as an energy source.

#### Proline

Proline is another important amino acid of the insect midgut [87]. It is oxidized by proline oxidase (or proline dehydrogenase) and δ-1-pyrroline-5-carboxylate dehydrogenase to glutamic acid [89], and both enzymes are predicted to be present in *B. nonstop* (Table S6).

None of the trypanosomatids possesses a gene for ornithine aminotransferase and, thus, they likely cannot form L-glutamate 5-semialdehyde, an intermediate in proline biosynthesis from ornithine. While Leishmaniinae are predicted to synthesize proline from glutamate using γ-glutamyl-phosphate reductase and γ-glutamyl kinase [69], the latter enzyme was not found in the genome of *B. nonstop* (Table S6). Since *T. cruzi* possesses a bifunctional pyrroline-5-carboxylate synthase that combines enzymatic activities of kinase and reductase and can convert glutamate to pyrroline-5-carboxylate directly [90], we have performed a focused search that readily revealed a gene encoding this bifunctional enzyme (annotated as γ-glutamyl-phosphate reductase) not only in *B. nonstop* (Table S6), but also in several other trypanosomatids. Thus, these parasites are all predicted to form proline from glutamate.

#### Glycine and histidine

**Fig. 2:**
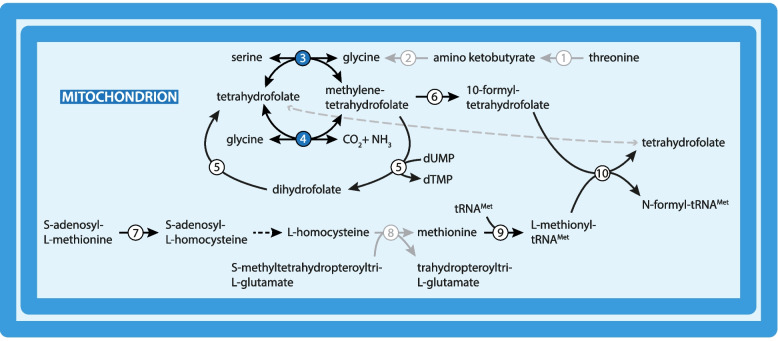
Serine-driven 1C and folate metabolism. Accession numbers of *B. nonstop* homologs are listed in Table S7. Numbers in colors represent proteins with predicted targeting signal (mitochondrial, blue; no signal; white). Numbers and arrows in light-grey represent enzymes that were not identified. Enzymes: 1, L-threonine 3-dehydrogenase; 2, glycine-C-acetyl transferase; 3, glycine cleavage system; 4; serine hydroxymethyltransferase; 5, dihydrofolate reductase-thymidylate synthase; 6, C-1-tetrahydrofolate synthase; 7, thiopurine S-methyltransferase; 8; 5-methyltetrahydropteroyltriglutamate-homocysteine S-methyltransferase; 9, methionyl-tRNA synthetase; 10, methionyl-tRNA formyltransferase

Glycine is split into CO_2_ and formic acid by the mitochondrial glycine cleavage system [91]. As in other kinetoplastids, genes for the subunits P, H, T, and L of this enzymatic complex were detected in the *B. nonstop* genome. Glycine is formed from serine by the action of serine hydroxymethyltransferase, which is usually present in two copies as the cytosolic and mitochondrial isoenzymes. The only identified sequence in *B. nonstop* possesses MTS, likely associating it with the organelle (Fig. 2; Table S7).

A full degradation pathway from histidine to glutamate comprising genes for histidine-ammonia lyase, urocanate hydratase, imidazolonepropionase, and formiminoglutamase is present only in two trypanosomatids, namely *Paratrypanosoma confusum* and *T. cruzi* [69, 92]. In other trypanosomatids, including *B. nonstop*, all four enzymes of this pathway have been lost (Table S6). Histidine cannot be formed *de novo* in any investigated trypanosomatid species.

#### Branched-chain amino acids: isoleucine, valine, and leucine

We predict that similarly to Leishmaniinae, the branched-chain amino acids isoleucine and valine in *B. nonstop* are first transaminated in the cytosol to their corresponding ketocarboxylic acids by a branched-chain aminotransferase. They are further oxidized in mitochondria *via* short/branched chain acyl-CoA dehydrogenase or isovaleryl-CoA dehydrogenase, followed by a hydratase, first to propionyl-CoA and finally *via* the methylmalonyl-CoA pathway to the TCA intermediates succinyl-CoA and acetyl-CoA. The latter two can either be used for the formation of acetate, a precursor for fatty acid synthesis, or *via* oxaloacetate/malate as substrates for gluconeogenesis [93]. Being equipped with all three genes of the methylmalonyl-CoA pathway (Fig. 1; Table 1; Table S6), *B. nonstop* in this respect resembles Leishmaniinae and differs from the American and African trypanosomes, *Blechomonas ayalai*, and *Phytomonas* spp.

Leucine is first converted to hydroxymethylglutaryl-CoA (HMG-CoA) by a pathway similar to that responsible for converting methionine. In the African trypanosomes, *P. confusum*, and *B. saltans*, the HMG-CoA is cleaved by HMG-CoA lyase into acetyl-CoA and acetoacetate [69]. However, similarly to Leishmaniinae, *B. nonstop* lacks the HMG-CoA synthase and HMG-CoA lyase genes (Table S6) allowing HMG-CoA to be directly incorporated into sterols *via* the isoprenoid synthetic pathway [94]. We conclude that no trypanosomatid is able to synthesize any of the three branched-chain amino acids *de novo*.

#### Lysine and serine

Same as other trypanosomatids, the predicted proteome of *B. nonstop* lacks any lysine degrading and *de novo* lysine synthesis capacity. Except for Leishmaniinae, all these flagellates also lack genes enabling them to convert diaminopimelate to lysine (Table S6).

In other trypanosomatids, serine is converted to cysteine *via* serine acetyltransferase and cysteine synthase, and then to pyruvate by cysteine desulfurase [95]_._ *Blastocrithidia nonstop* is predicted to share this pathway with most trypanosomatids, except for the African trypanosomes and *Phytomonas* spp. (Table S6). The first enzyme of serine synthesis, D-3-phosphoglycerate dehydrogenase, is present in Leishmaniinae, but not in the *B. nonstop* genome, while the second enzyme, phosphoserine phosphatase, was not detected in any kinetoplastid (Table S6). Thus, *B. nonstop* is predicted to not synthesize serine *de novo*.

#### Methionine

Methionine is an important substrate for methylation and formation of polyamines. Its degradation involves a pathway consisting of eight steps (Table S6). Methionine is first transaminated in the cytosol to α-ketobutyrate, after which it is oxidized in the mitochondria to succinyl-CoA. This situation, first reported in *Leishmania* spp. [10, 96], was later extended to other members of Leishmaniinae, *B. saltans*, and *P. confusum*. In the remaining trypanosomatids, methionine cannot be oxidized beyond the stage of propionyl-CoA [69]. As mentioned above, the methylmalonyl-CoA pathway is predicted to be operational in *B. nonstop*.

In contrast to Leishmaniinae [10], *B. nonstop* appears unable to synthesize methionine *de novo*. It lacks both genes for two methionine synthase isoenzymes, namely cobalamine-dependent and cobalamine-independent methionine synthases. However, similar to all other trypanosomatids, methionine can likely be salvaged by *B. nonstop* (all eight enzymes of the methionine-recycling pathway were found in the predicted proteome of this species) (Table S6).

#### Threonine

In trypanosomatids, there are two major routes for degradation of L-threonine [11]. In the first route, threonine is catabolized by threonine dehydrogenase to form L-2-amino-3-oxobutanoate, which is cleaved by 2-amino-3-ketobutyrate-CoA ligase, forming glycine and acetyl-CoA. In the second route, threonine is catabolized by threonine dehydratase to ammonia and α-ketobutyrate, which is irreversibly converted to propionyl-CoA and formate. Notably, both pathways appear to be absent in *B. nonstop*, as genes for threonine dehydrogenase and serine threonine dehydratase were not found in the genome of the species under analysis (Table S6). A metabolic alternative to the pathways described above is the cleavage of threonine into acetaldehyde and glycine by serine hydroxymethyltransferase [97–99]. In most trypanosomatids, threonine is formed from homoserine rather than from aspartate, and this also appears to be the case for *B. nonstop*, which is predicted to possess homoserine kinase and threonine synthase responsible for threonine formation (Table S6).

#### Aromatic amino acids

The classical aerobic pathway of aromatic amino acid oxidation appears absent in *B. nonstop*. However, all three enzymes of the anaerobic degradation pathway, i.e., indole-3-pyruvate decarboxylase, tyrosine aminotransferase, and aspartate aminotransferase [69] were identified in the predicted *B. nonstop* proteome (Table S6). This suggests that phenylalanine is converted to phenylacetate, as has been reported previously for some other trypanosomatids [100, 101].

Apart from *B. saltans* and *P. confusum*, the entire pathway of tryptophan degradation has been lost in all other members of Trypanosomatidae, including *B. nonstop*, as can be judged from the predicted proteome. This has been explained by adaptation of these organisms to their parasitic lifestyle, where the need for amino acids-derived carbon is largely compensated for by an abundance of glucose and other fermentable substrates from their hosts [24].

### Serine-driven 1C, folate, and biopterin metabolism

Serine-driven one-carbon (1C) metabolism is essential for methylation and production of intracellular reduced NADPH. In this pathway, serine is converted to glycine by the action of both cytosolic and mitochondrial serine hydroxymethyltransferases. In the mitochondrion, glycine is converted to ammonia and CO_2_ by the glycine cleavage system [69, 87]. The latter pathway is operational in most trypanosomatids. This now can be extended to *B. nonstop*, which is predicted to possess relevant enzymes (Fig. 2; Table S7). While *L. major* encodes genes for both cytosolic and mitochondrial serine hydroxymethyltransferases, *B. nonstop* appears to have only the mitochondrial isoenzyme producing methylenetetrahydrofolate, which then fuels the 1C metabolism and is involved in generation of the final formyl-tRNA^Met^. Although the predictions do not suggest mitochondrial localization (Table S7), the whole pathway was recently experimentally localized to the mitochondrion of *T. brucei* [102]. Thus, precise localization of these enzymes in *B. nonstop* needs to be investigated further.

All trypanosomatids are folate/pterin auxotrophs, i.e., they are unable to synthesize their own folates, such as folic acid and biopterin [103–105]. To compensate for this, they salvage exogenous folates from their hosts. *Leishmania* spp. have been shown to carry biopterin transporters, as well as pterin reductase 1 [10, 21, 106, 107]. A comparison of enzymes involved in folate metabolism between the model *L. major* and *B. nonstop* (based on the genome) revealed an important deficit in biopterin metabolism in the latter. Although the presence of six homologous members of the biopterin/pterin/folate transporter family confirms the importance of folate metabolism in *B. nonstop*, six out of 22 enzymes of folate/biopterin metabolism appear to be absent (Table S7). PTR1 (methotrexate resistance factor), which is involved in the formation of both H4-biopterin and tetrahydrofolate, is missing, as well as the quinonoid dihydropteridine reductase and biopterin-dependent phenylalanine-4-hydroxylase. As such, biopterin likely cannot be converted into H4-biopterin-4-α-carbinolamine in *B. nonstop* . Other predicted deficiencies of folate metabolism in *B. nonstop* are the absence of methylenetetrahydrofolate reductase and two iso-functional enzymes of cobalamin-dependent and -independent methionine synthase, implying that *B. nonstop* is dependent on a source of external folate. The absence of PTR1, which also functions as a back-up of the dihydrofolate reductase, suggests that contrary to other trypanosomatids, *B. nonstop* is sensitive to the antifolate methotrexate.

### Urea cycle, polyamine biosynthesis, and energy storage

Leishmaniinae uniquely acquired several enzymes of the urea cycle by HGT [11], yet in the genome of *B. nonstop* this cycle is clearly absent, since genes for arginase, argininosuccinate synthase, and argininosuccinate lyase were not detected (Table S8). The non-proteinogenic amino acid ornithine is decarboxylated into putrescine and used for polyamine biosynthesis [108]. The responsible enzyme, ornithine decarboxylase is present in *B. nonstop*, while it has not been found in any Leishmaniinae (Table S8). Similar to Leishmaniinae, the predicted proteome of *B. nonstop* has a lysine decarboxylase (Table S6) that can decarboxylate both diaminopimelic acid and lysine to the polyamine cadaverine (1,5-pentadiamine).

Creatine phosphate, used as an energy storage by many eukaryotes [109], is absent in all trypanosomatids [110]. Arginine phosphate is an alternative energy-rich molecule, formed by arginine kinase replacing creatine phosphate in some eukaryotes. Arginine kinase is present not only in the free-living *B. saltans*, but in most trypanosomatids [111, 112], including *B. nonstop* as judged from its predicted proteome (Table S6), while it is missing from Leishmaniinae and *P. confusum* [69].

### Metabolism of nucleotides

#### Purine and pyrimidine biosyntheses

The absence of purine biosynthetic pathway is frequent in parasites including trypanosomatids [24]. Only one (adenylosuccinate lyase) out of ten enzymes of the purine biosynthetic pathway was identified in the *B. nonstop* genome (Table S9). However, since this enzyme also plays an important role in the purine salvage as part of the purine nucleotide cycle (see below), the examined protist can apparently acquire all the essential purines directly from the host.

A complete operon structure containing all five *Pyr* genes involved in the synthesis of uridine monophosphate from ammonia, CO_2_, aspartate and ribose, the building blocks of pyrimidine nucleotides, have been found in all trypanosomatids [113], including *B. nonstop* (Table S9).

#### Purine salvage

All enzymes for the interconversion of purine bases and nucleosides are present in the predicted *B. nonstop* proteome (Table S9) indicating that the purine salvage mechanisms [81] may be operational. Genes for the three enzymes of purine nucleotide cycle, i.e., adenylosuccinate synthetase, adenylosuccinate lyase, and AMP deaminase, are present as well. Other genes for enzymes of purine metabolism, such as hypoxanthine-guanine phosphoribosyltransferase, inosine monophosphate dehydrogenase, GMP synthase, GMP reductase, adenine phosphoribosyltransferase, ribose kinase, phosphoribosylpyrophosphate synthase, purine-specific nucleoside hydrolase (inosine-, adenosine-, guanosine-nucleoside hydrolase), and adenosine kinase, were all detected (Table S9). The majority of them have previously been shown to be associated with glycosomes [114]. This seems also to be the case for *B. nonstop* since several of them harbor the PTS1 sequences.

### Synthesis of sugar nucleotides

Sugar nucleotides are the activated forms of monosaccharides [115]. They function as glycosyl donors in numerous glycosylation reactions. The resulting glycoconjugates, expressed on the surface of trypanosomatid parasites, fulfil a vital role in the host-parasite interaction and are essential for infectivity, virulence, and parasite survival inside the host [116]. An inventory of the enzymes potentially involved in sugar nucleotide biosynthesis present in *B. nonstop* shows that several of them are missing (Table S10) including phosphoglucomutase, which is also absent in the African trypanosomes [117]. In the latter species, this activity is substituted by two other enzymes, phosphomannomutase and phospho-N-acetylglucosamine mutase, and genes encoding both these enzymes are present in *B. nonstop*. The absence of UDP-glucose 4,6-dehydratase can be compensated by the presence of UDP-galactose 4-epimerase, suggesting that galactofuranose can be incorporated into glycoconjugates. The predicted absence of fucose/arabinose kinase and fucose/arabinose kinase/pyrophosphorylase suggests that *B. nonstop* is unable to incorporate arabinose in its glycoconjugates. This property is shared with all other trypanosomatids, except for Leishmaniinae [69]. However, the fucose synthesis and its incorporation appears operational in *B. nonstop* (Table S10), since it encodes the necessary enzymes, i.e., GDP-mannose pyrophosphorylase, GDP-mannose 4,6-dehydratase, GDP-L-fucose synthetase, and fucosyl transferase. This pathway was recently experimentally localized to the mitochondrion of *T. brucei* [102], even though these proteins lack obvious targeting signals (Table S10).

### RNA interference

Three of the four genes (argonaute, dicer, and PIWI), which in *T. brucei*, *L. pyrrhocoris*, and *C. fasciculata* have been identified as essential for RNA interference [118, 119], are also present in the *B. nonstop* genome (Table S11) suggesting that the pathway is functional. However, a homolog of dicer DCL2 was not found.

### Lipid metabolism

The predicted proteome of *B. nonstop* has a complete set of enzymes for the synthesis and degradation of lipids [69, 120, 121] (Table S12). Triglycerides can likely be split in glycerol and fatty acids by triglyceride lipases as detailed below.

#### Fatty acid degradation

The free fatty acids are activated by several acyl-CoA synthetases of different chain length specificity and subsequently shortened by β-oxidation to acetyl-CoA by acyl-CoA dehydrogenases of different length specificity [122, 123]. In *B. nonstop*, the process of β-oxidation may take place in both the mitochondrion and glycosomes, as can be inferred from the presence of both mitochondrial acyl-CoA dehydrogenase and glycosomal acyl-CoA oxidase and glycosomal bifunctional enzyme in the predicted proteome of the species (Fig. 1; Table 1). Although termed bifunctional, the latter enzyme combines three functions of enoyl-CoA isomerase, enoyl-CoA hydratase, and 3-hydroxyacyl dehydrogenase [33]. While we identified only the supposedly mitochondrial 3-ketoacyl-CoA thiolase, we cannot rule out its dual localization in both the mitochondrion and glycosomes, as was recently experimentally confirmed in *T. brucei* for the bifunctional enzyme [102].

#### Fatty acid synthesis

Cytosolic fatty acid synthesis in trypanosomatids differs from that found in most other eukaryotes because acetyl-CoA, after being carboxylated to malonyl-CoA, is used in a series of fatty acid elongation reactions driven by the fatty acid elongases, instead of the canonical cytosolic multi-subunit fatty acid synthase complex of type I [124–126]. The predicted proteome of *Blastocrithidia nonstop* carries a complete battery of fatty acid elongases (Fig. 1; Table 1), as well as several fatty acid desaturases (Table S12).

The pathway for unsaturated fatty acid biosynthesis has been shown to be essential for trypanosomatid parasites [127]. As one of their representatives, *B. nonstop* appears able to make its own ether lipids. The three enzymes of this pathway (alkyl-dihydroxyacetone phosphate synthase, 1-acyl-sn-glycerol-3-phosphate acyltransferase, and alkyl-dihydroxyacetone phosphate acyltransferase) [121] are present in the predicted proteome of this species and two of them carry the PTS1 (Table S12) suggesting a glycosomal localization of this pathway.

#### Mevalonate pathway and sterol biosynthesis

All enzymes of the mevalonate pathway and sterol biosynthesis are present in the predicted proteome of *B. nonstop* (Table S13, Fig. S1). Moreover, the enzymatic targets for some of the anti-fungal azoles could be identified as well. Miconazole, ketaconazole and itraconazole are shown to inhibit sterol 14-α-demethylase CYP51, a member of the cytochrome P450 family that catalyzes conversion of lanosterol to ergosterol [128, 129]. CYP51 is present in the predicted proteome of *B. nonstop* (Table S13).

#### Phospholipids and phospholipases

While the free-living *B. saltans* is endowed with 23 different phospholipases and five lysophospholipases [69], *B. nonstop* proteome appears to lack phospholipase B and retain a very limited repertoire of one phospholipase A2, three distinct copies of phosphoinositol-specific phospholipase C, and only a single lysophospholipase (Table S14).

### Vitamins

An inventory of the enzymes involved in the synthesis and utilization of vitamins and cofactors in the predicted proteome of *B. nonstop* revealed that this species is likely auxotrophic for all the vitamins, except vitamin C (Table S15). While its genome lacks ascorbate peroxidase, the presence of a gene for an iron/ascorbate oxidoreductase indicates that the parasite may be able to synthesize its own vitamin C. This ascorbate pathway is of the yeast-type, in which prostaglandin f2-α synthase reduces arabinose to arabinolactone, which is then converted to ascorbate [10, 130].

### Heme and iron uptake system

As an essential growth factor of trypanosomatids [131], heme is required for the synthesis of heme-containing proteins, such as cytochromes and catalase [132]. *Blastocrithidia nonstop* has acquired the last three genes of the heme biosynthetic pathway (protoporphyrinogen oxidase, coproporphyrinogen III oxidase, and ferrochelatase) by HGT from bacteria (Table S16). The early enzymes of the heme biosynthesis pathway appear to be absent.

A homolog of the heme receptor LHR1 previously described in *Leishmania* spp. and lacking homologs outside of this group [133–135] is also found in the predicted proteome of *B. nonstop*. Due to the presence of other genes involved in heme and iron uptake in this species (Table S16), it, as all investigated trypanosomatids, is predicted to be a heme auxotroph that must assimilate heme either from its host or from the culture medium [10, 136].

Most trypanosomatids (except *Phytomonas* spp.) have a copy of a protoheme IX farnesyltransferase that converts heme *b* to heme *a*. This gene (Table S16) is indicative of the presence of a classical respiratory chain with a cyanide-sensitive cytochrome aa3-containing cytochrome oxidase in *B. nonstop*. We validated this prediction experimentally (Fig. S2).

## Conclusions

In this work, we aimed to predict the metabolic capacity of *Blastocrithidia nonstop*, a trypanosomatid with all three stop codons turned into sense codons*.* We hypothesized that such a dramatic departure from the conventional genetics might also have an impact on metabolism. Indeed, the frequency of in-frame stops appears to negatively correlate with the expression of a given gene. We hypothesized that because of this, some metabolic pathways may run slowly or some pathways (or their parts) may get ablated reflecting a burden imposed on their components by the accumulated in-frame stop codons. Rather unexpectedly, we found that (unlike its genetic code) the metabolism of *B. nonstop* did not deviate much from its kin in related flagellates.

## Methods

### Cultivation of *Blastocrithidia nonstop*

*Blastocrithidia nonstop* was cultivated in a semi-defined Schneider's *Drosophila* medium (Merck, St. Louis, USA) supplemented with 2 μg/ml hemin (BioTech, Prague, Czechia), 25 mM HEPES pH 7.5, 100 units/ml of penicillin, 100 μg/ml of streptomycin (all from VWR, Radnor, USA), and 10% Fetal Bovine Serum (Termo Fisher Scientifc, Waltham, USA) as in [19]. Species identity was confirmed by amplifying and sequencing 18S rRNA gene as described previously [137, 138].

### Immunofluorescence microscopy

Cells were processed as described previously [139]. In brief, cells on glass slides were fixed with 4% (w/v) paraformaldehyde for 15 minutes, permeabilized with 1% (v/v) NP-40 for 20 minutes, and blocked with 1% (w/v) bovine serum albumin (BSA) for 1 hour. All the above steps were performed at room temperature using reagents from Sigma-Aldrich/ Merck, Burlington, USA. After three washes with phosphate buffered saline (PBS), slides were stained with rabbit polyclonal anti-phosphomevalonate kinase (MVAK, gift from D. González-Pacanowska to V.Y.) [36] or anti-triosephosphate isomerase (TIM, gift from P. Michels to J.L.) [38, 140] antibodies (both at 1:1,000 in PBS with 0.1% BSA and 0.1% Tween-20; Sigma-Aldrich/ Merck), and visualized with goat anti-rabbit CF488A-conjugated antibody (1:10,000) (Sigma-Aldrich/ Merck) in the same buffer. The nucleus and kinetoplast DNA were stained with 4′,6-diamidino-2-phenylindole (DAPI; Sigma-Aldrich/ Merck). Images were acquired on Olympus BX53 fluorescent microscope (Olympus, Tokyo, Japan) equipped with the Olympus DP72 camera and processed in ImageJ Fiji v2.14.0 [141]. The experiments were performed three times; representative images are presented in Fig. S1.

### Respiration analysis

*Blastocrithidia nonstop* cells in the log phase of growth were harvested by centrifugation at 1,500 × g for 5 minutes and washed in MiRO5 medium (Oroboros Instruments, Innsbruck, Austria). Oxygen uptake was monitored by the Oroboros FluoRespirometer (Oroboros Instruments). To assess the basal cell respiration, 5 × 10^7^ cells were placed into the chamber and monitored for 5 minutes. Next, *in situ* digitonin titration was performed to obtain the intact mitochondria. When the oxygen rate [pmol of O_2_/cell/s] was close to zero, respiration was initiated by the addition of 10 mM of succinate (a substrate for complex II) and 2.5 mM of ADP (to increase the rate of respiration). Cytochrome *c* (10 mM) was used to control mitochondrial membrane integrity, the protonophore uncoupler (carbonyl cyanide m-chlorophenyl hydrazine) at 0.5 mM was used to control maximal capacity of oxygen uptake, and malonic acid at 5mM was added as a specific inhibitor of complex II. All chemicals were from Sigma-Aldrich/ Merck. The increase in oxygen uptake was monitored in three biological replicates; a representative profile is presented in Fig. S2.

### Bioinformatic analyses

Predicted protein sequences from *B. nonstop* [27] were annotated by blastp searches (with a cut-off *e*-value of 10^-20^ [142]) against protein datasets of a selection of kinetoplastids available from TriTrypDB (release 64) [15] including *Angomonas deanei*, *Blechomonas ayalai*, *Bodo saltans*, *Crithidia fasciculata*, *Endotrypanum monterogeii*, *Leishmania major* Friedlin, *Leishmania martiniquensis*, *Leishmania mexicana*, *Leptomonas pyrrhocoris*, *Leptomonas seymouri*, *Paratrypanosoma confusum*, *Porcisia hertigi*, *Trypanosoma brucei* TREU927, *Trypanosoma cruzi* CL Brener Esmeraldo-like. Proteins not retrieving any hit were then screened (with a cut-off *e*-value of 10^-5^) against the Swiss-Prot database (downloaded February 10, 2023) [143]. Protein domains were further annotated using InterProScan v5.55-88.0 [144] and the Pfam database [145]. Annotated protein dataset of *B. nonstop* is available from Figshare under the link: https://doi.org/10.6084/m9.figshare.24064443.

To identify proteins involved in the *B. nonstop* metabolism, annotated proteins were used as query in “all against all” blastp searches (with a cut-off *e*-value of 10^-20^ [142]) against the proteomes of selected trypanosomatids available from the TriTrypDB. In some cases, protein sequences from the free-living *B. saltans* [69] and the diplonemid *Paradiplonema papillatum* [146] were used to search the *B. nonstop* proteome. In case of the missing proteins, they were searched for in the genome of *B. nonstop* [27].

Peroxisomal targeting sequences were identified by searching the *B. nonstop* proteome as described previously [33]. The PTS1 was defined as [SAGCNP]-[RHKSNQ]-[LIVFAMY]$ and the PTS2 as ^M-x{1,10}-[RK]-[LVI]-x{5}-[HQ]-[ILA]. Mitochondrial predictions were performed by TargetP v2.0 [147] or by an in-house search of the following pattern: ^M-[RHKFL]-x{0,1}-[RKHST]-x{1,10}-[STRK].

### Supplementary Information

**Additional file 1:** **Fig. S1.** Glycosomes of B. nonstop. Glycosomal markers triosephosphate isomerase (TIM; glycolysis) and mevalonate kinase (MVK; sterol biosynthesis) were stained with their respective antibodies to visualize glycosomes. Nuclei and kinetoplasts were stained with DAPI. BF, bright field. Scale bar, 10 μm. **Fig. S2.** Respiration in B. nonstop. oroboros measurements of oxygen flow in digitonin-permeabilized. *B*. nonstop cells after stimulation with succinate. Other added chemicals are indicated on top. Blue and red lines represent oxygen concentration in μM and oxygen flow (oxygen consumed per cell per second), respectively.

**Additional file 2:** **Table S1.** Glycosomes and glycolysis-related proteins. **Table S2.** Acidocalcisomes. **Table S3.** Mitochondrial translocation machinery. **Table S4.** OXPHOS subunits and ubiquinone synthesis. **Table S5.** Mitochondrial metabolism. **Table S6.** Amino acid metabolism. **Table S7.** Serine-driven 1C, folate, and biopterine metabolism. **Table S8.** Urea cycle. **Table S9.** Pyrimidine biosynthesis and purine salvage. **Table S10.** Synthesis of sugar nucleotides. **Table S11.** RNA interference. **Table S12.** Lipid metabolism. **Table S13.** Mevalonate and sterol synthesis. **Table S14.** Phospholipids and phospholipases. **Table S15.** Vitamins and cofactors. **Table S16.** Heme biosynthesis and iron uptake system.

## Data Availability

Annotated protein dataset of *B. nonstop* is available from Figshare under the link: https://doi.org/10.6084/m9.figshare.24064443.
