# Supplementary material for: In silico prediction of the metabolism of Blastocrithidia nonstop, a trypanosomatid with non-canonical genetic code
Source: BMC Genomics. 2024 Feb 16;25:184. doi: 10.1186/s12864-024-10094-8 (PMC10874023; doi:10.1186/s12864-024-10094-8)
Supplement: Supplementary file 1 — Additional file 1: Fig. S1. Glycosomes of B. nonstop. Glycosomal markers triosephosphate isomerase (TIM; glycolysis) and mevalonate kinase (MVK; sterol biosynthesis) were stained with their respective antibodies to visualize glycosomes. Nuclei and kinetoplasts were stained with DAPI. BF, bright field. Scale bar, 10 μm. Fig. S2. Respiration in B. nonstop. oroboros measurements of oxygen flow in digitonin-permeabilized. B. nonstop cells after stimulation with succinate. Other added chemicals are indicated on top. Blue and red lines represent oxygen concentration in μM and oxygen flow (oxygen consumed per cell per second), respectively. [file 12864_2024_10094_MOESM1_ESM.pdf]

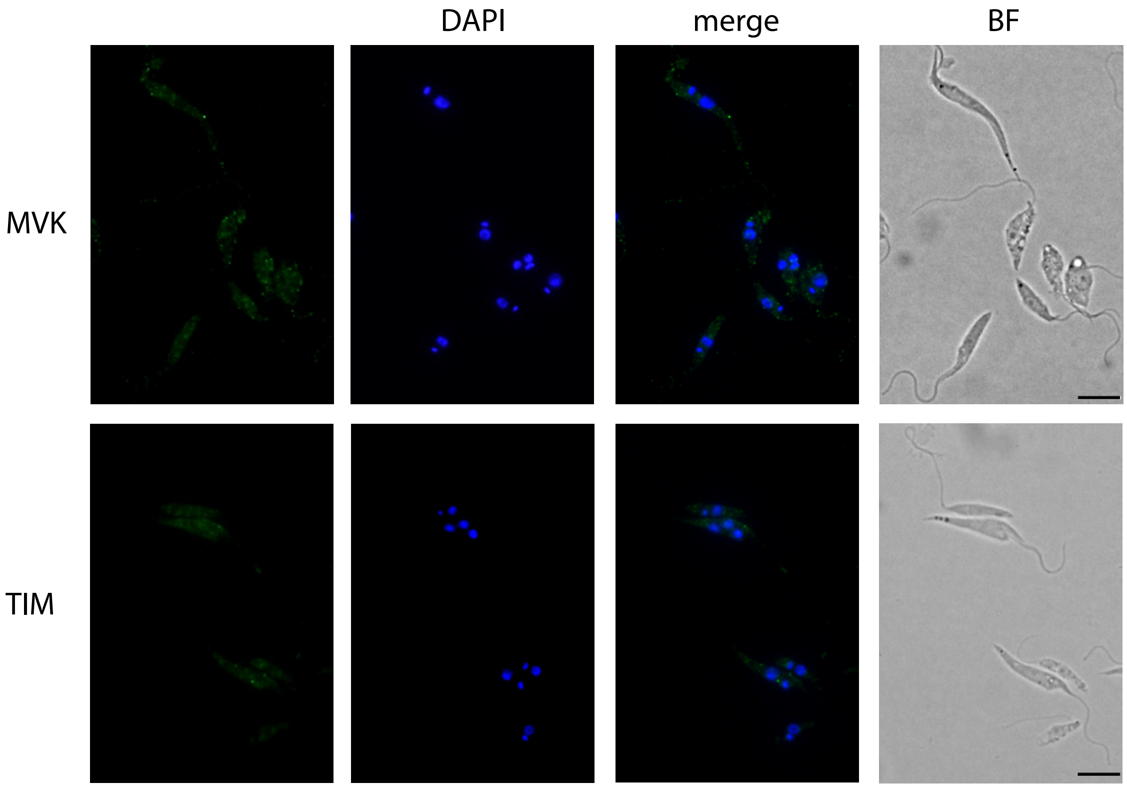

Fig. S1. Glycosomes of *B. nonstop*. Glycosomal markers triosephosphate isomerase (TIM; glycolysis) and mevalonate kinase (MVK; sterol biosynthesis) were stained with their respective antibodies to visualize glycosomes. Nuclei and kinetoplasts were stained with DAPI. BF, bright field. Scale bar, 10 µm.

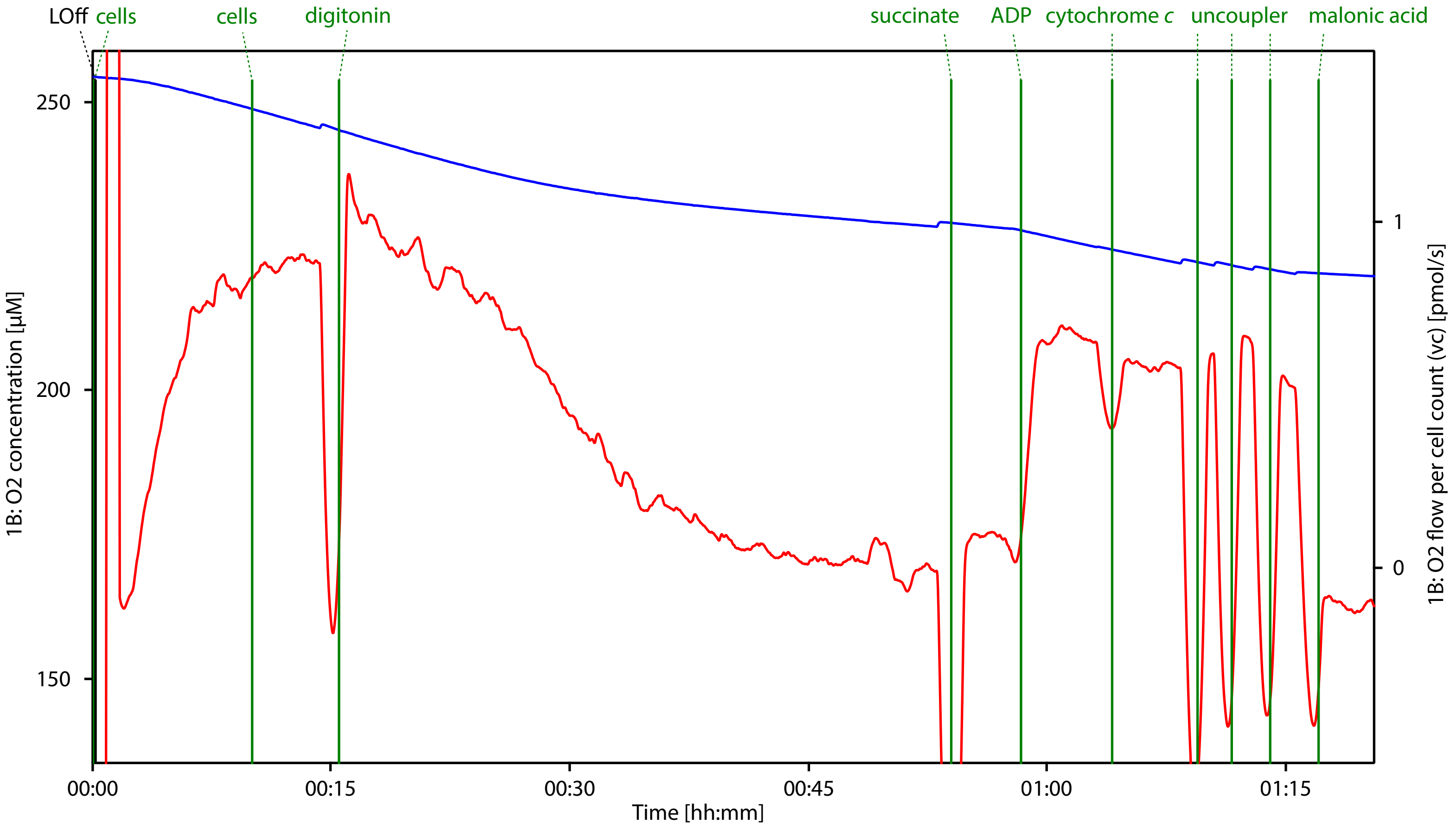

Fig. S2. Respiration in *B. nonstop*. Oroboros measurements of oxygen flow in digitonin-permeabilized *B. nonstop* cells after stimulation with succinate. Other added chemicals are indicated on top. Blue and red lines represent oxygen concentration in µM and oxygen flow (oxygen consumed per cell per second), respectively.
